# Editorial: Lignin valorization: Recent trends and future perspective

**DOI:** 10.3389/fbioe.2023.1190128

**Published:** 2023-04-11

**Authors:** Daochen Zhu, Bin Yang, Hongliang Wang, Mohd. Shahnawaz

**Affiliations:** ^1^ Biofuels Institute, School of Emergency Management, School of the Environment and Safety Engineering, Jiangsu University, Zhenjiang, Jiangsu, China; ^2^ Biological Systems Engineering and the Bioproduct Sciences and Engineering Laboratory, Washington State University, Richland, WA, United States; ^3^ Center of Biomass Engineering/College of Agronomy and Biotechnology, China Agricultural University, Beijing, China; ^4^ Department of Botany, University of Ladakh, Kargil, India

**Keywords:** lignin valorization, bioconversion, pathway, chemicals, depolymerization

Lignin is a complex and heterogeneous material and regarded as the second-most prevalent biopolymer on the planet. It is reported as a waste product from the pulp and paper industry (Tribot et al., 2019). However, recent research efforts have focused on finding ways to convert lignin into valuable products and thereby promote its use as a sustainable feedstock for various applications (Yan et al., 2021).

This Research Topic of Frontiers in Bioengineering and Biotechnology presents a Research Topic of papers that highlight the latest advances in lignin valorization. The four papers cover a diverse range of Research Topic and approaches, including biorefinery processes, endophytes, genetic engineering, and microbial biotechnology.

The first paper by Chen et al. discusses the integrated cascade biorefinery process to convert woody biomass into phenolic monomers and carbon quantum dots. The process involves two stages: the first stage uses an acidic aqueous solution to extract lignin from woody biomass, which is then further processed to produce phenolic monomers; the second stage converts the remaining biomass into carbon quantum dots through hydrothermal carbonization. The authors demonstrate that this two-stage process can achieve high yields of both phenolic monomers and carbon quantum dots while also reducing waste and environmental impact. They also suggest that the process could be used as a model for other integrated cascade biorefinery processes to produce a range of high-value products from biomass. The paper provides detailed information on the experimental setup and results, including analysis of the chemical composition of the extracted lignin and the properties of the resulting phenolic monomers and carbon quantum dots. The authors also discuss potential applications for these products, such as in the production of adhesives, coatings, and electronic devices.

The second paper by Mattoo and Nonzom explores the potential of endophytic fungi to be used in the valorization of lignin, which is a complex organic polymer found in plants. After cellulose, lignin is the second most prevalent polymer in nature and makes up a significant portion of plant cell walls. Lignin is a challenging material to degrade and thus, its valorization is a promising approach towards sustainability. Fungi that dwell inside plant tissues without harming their host plants are known as endophytic fungi. They have been studied for their potential to produce bioactive compounds, but their potential for lignin valorization is largely unexplored. The authors suggest that endophytic fungi can be used to produce ligninolytic enzymes, which can break down lignin into smaller molecules that can be used as precursors for the production of biofuels, chemicals, and other value-added products. The paper provides an overview of the ligninolytic enzymes produced by endophytic fungi and their potential applications. The authors conclude that endophytic fungi have the potential to be a valuable tool in lignin valorization and that further research is needed to fully understand their potential and optimize their use.

The third paper by Wang et al. examine the potential of spatio-temporal manipulation of lignin production in plants in order to enhance lignocellulose and valorize lignin. Although lignin, a complex organic polymer, is essential for the composition and durability of plant cell walls, it presents difficulties for the effective use of plant biomass.

The authors suggest that plants’ lignin biosynthesis may be modified spatiotemporally to improve lignocellulose by reducing the recalcitrance of lignin, enhancing enzymatic hydrolysis, and increasing the accessibility of cellulose. The paper provides an overview of recent advances in lignin biosynthesis regulation, including transcriptional and post-transcriptional modifications, as well as genetic engineering approaches for spatiotemporal manipulation of lignin production.

Together with possible uses in plant breeding for the production of bioenergy and biomaterials, the authors also cover the prospective applications of spatiotemporal modulation of lignin biosynthesis and lignin valorization for the generation of high-value compounds and materials.

The fourth paper by Zhang et al. describes a study in which the yeast species Kluyveromyces marxianus was engineered to produce xylitol from corncob, a readily available lignocellulosic biomass. The researchers used genetic engineering techniques to introduce genes from other organisms involved in xylitol production, resulting in a strain of K. marxianus that was able to produce high yields of xylitol from corncob. The researchers optimized various parameters such as temperature, pH, and initial substrate concentration to obtain maximum xylitol production. They also investigated the effect of inhibitors present in corncob hydrolysate on xylitol production and found that the engineered strain was more tolerant to these inhibitors than the wild-type strain. The study demonstrated that corncob could be an efficient and sustainable feedstock for xylitol production using engineered K. marxianus. Xylitol has various industrial applications, including as a sweetener in food products and as a precursor for the generation of chemicals and materials. The use of lignocellulosic biomass as a feedstock for xylitol production has the potential to reduce the reliance on fossil fuels and contribute to a more sustainable and circular economy.

Overall, this Research Topic provides a comprehensive overview of the current state of lignin valorization research and highlights the challenges and opportunities associated with the use of lignin as a sustainable feedstock for various applications. We anticipate that this Research Topic of papers will stimulate new lignin valorization research and technological advancements, aid in the creation of more sustainable and circular bioeconomies, and do so.

We appreciate all the contributing authors of this Research Topic as well as the reviewers who offered insightful criticism. We would also like to acknowledge the support of the Frontiers in Bioengineering and Biotechnology editorial team for their assistance in publishing this Research Topic.

## References

[B1] Tribot A. Amer G. Abdou Alio M. de Baynast H. Delattre C. Pons A. (2019). Wood-lignin: Supply, extraction processes and use as bio-based material. Eur. Polym. J. 112, 228–240. 10.1016/j.eurpolymj.2019.01.007

[B2] Yan Z. Song B. Fang G. Wu T. Chen N. Zhao M. (2021). Bringing material concepts into conventional biorefineries: Considerations of sources, preparations, and applications of lignin nanomaterials. ACS Sustain. Chem. Eng. 9, 10403–10423. 31. 10.1021/acssuschemeng.1c02954

